# Supplementary material for: A new method for obtaining bankable and expandable adult-like microglia in mice
Source: J Neuroinflammation. 2021 Dec 17;18:294. doi: 10.1186/s12974-021-02351-4 (PMC8680120; doi:10.1186/s12974-021-02351-4)
Supplement: Supplementary file 1 — Additional file 1: Figure S1. Comparison of the cell yields according to the region and embryo stage. Flow cytometry revealed that the dissected head neuroepithelial layer (NEL) from mouse E13.5 could yield a higher ratio of CD11b-positive cells than the brain cortex separated from an identical mice group. Immunofluorescence showed that 13.5 NEL have a higher number of IBA-1-positive cells than neuroepithelial layer from mouse E9.5 or E17.5, when cultured for 21 days. Scale bar = 50 μm. Figure S2. Comparison of phagocytic activity among BV2, neonatal microglia, NEL-MG, and adult microglia. To assess the phagocytic activity, BV2, NEL-MG, neonatal microglia, and adult microglia at a density of 2 × 10^5^ cells/mL were seeded on a 12 mm coverslip in 24-well cell culture dishes. Each souce of micorglia were treated with 2 μL of red fluorescent latex beads (2 μm, Sigma-Aldrich, St. Louis, MO, USA). NEL-MG showed highest phagocytic activity among them. The data are shown as mean ± standard error of the mean (SEM). Statistical analysis was indicated as a table (below). Figure S3. Mass production of microglial cells via NEC passage culture. Our method produced thirty times the number of microglial cells than that of neonatal microglia when we used a cutoff of 100 days. The data are shown as mean ± standard error of the mean (SEM). Figure S4. Scatter plot comparison between adult microglia and NEL-MG or neonatal microglia (MG) Diagonal line indicates no significant difference between the two groups (fold change = 1) and the intensity values are normalized to the log2 transformed expression value. Figure S5. Mix with neurospheroid. CellTracker-tagging NEL-MG (red) were mixed with neurospheroids (NS) at different ratios and times. NEL-MG were mixed evenly with NS when we co-mixed them. IBA-1 can label both resident microglia and mixed NEL-MG. Table S1. List of microglial signature genes. Table S2. Primers information. [file 12974_2021_2351_MOESM1_ESM.docx]

**Additional file 1**

**
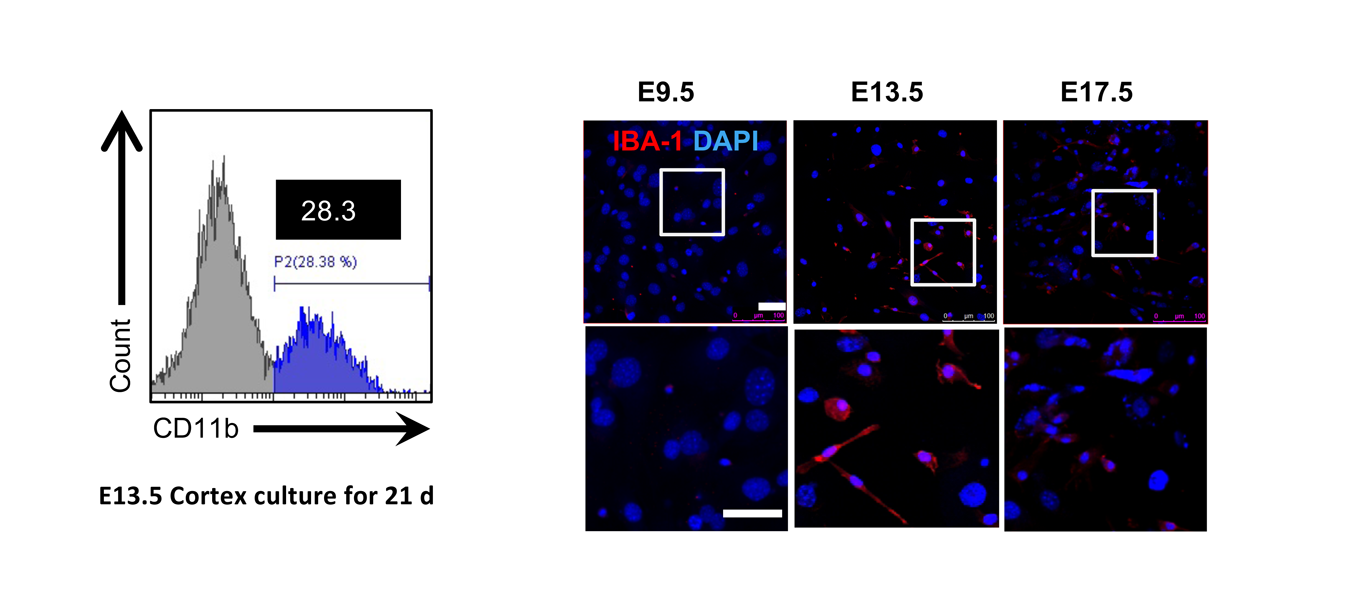
**

**Figure S1. Comparison of the cell yields according to the region and embryo stage**

Flow cytometry revealed that the dissected head neuroepithelial layer (NEL) from mouse E13.5 could yield a higher ratio of CD11b-positive cells than the brain cortex separated from an identical mice group. Immunofluorescence showed that 13.5 NEL have a higher number of IBA-1-positive cells than neuroepithelial layer from mouse E9.5 or E17.5, when cultured for 21 days. Scale bar = 50 μm.


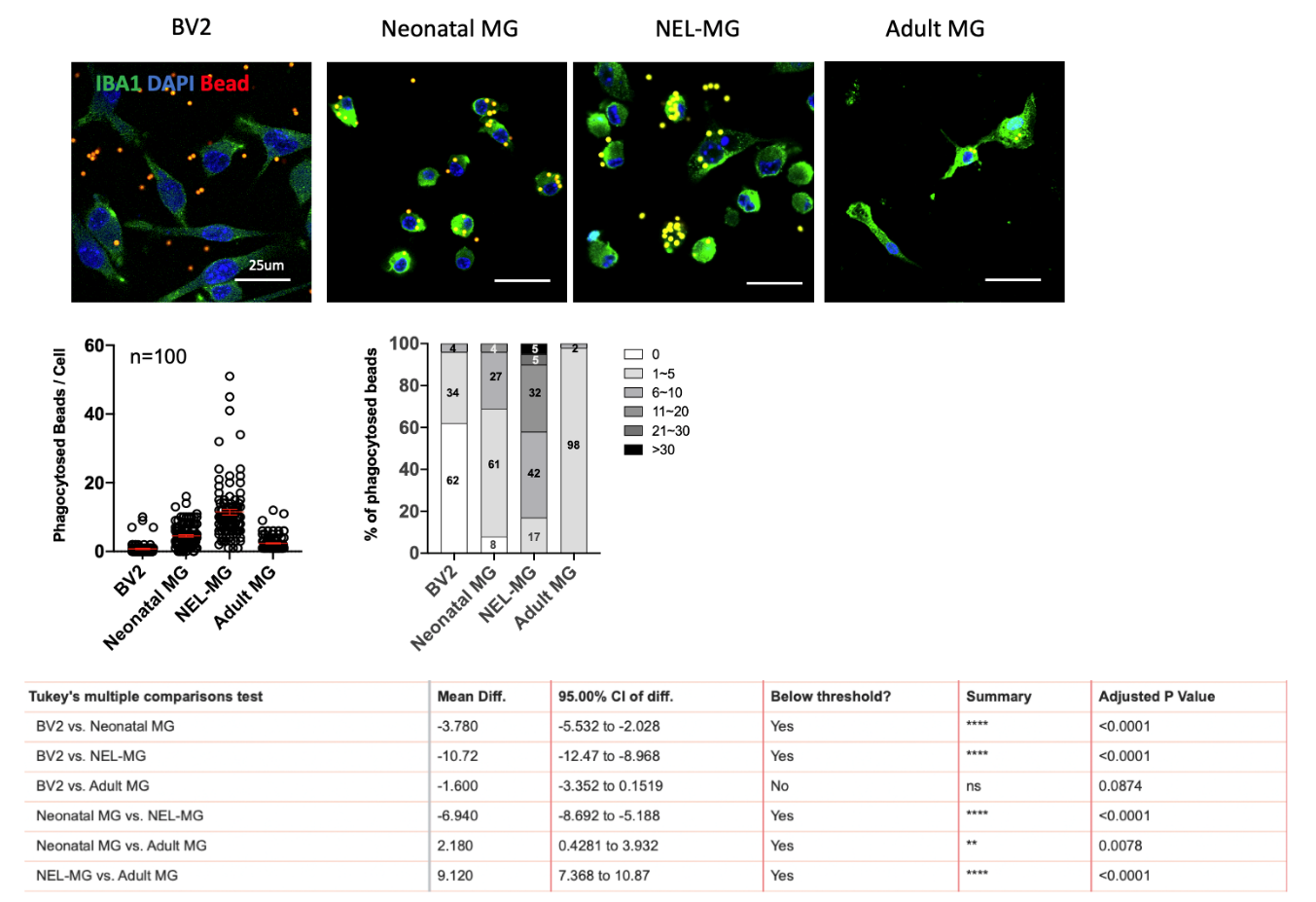


**Figure S2. Comparison of phagocytic activity among BV2, neonatal microglia, NEL-MG, and adult microglia**

To assess the phagocytic activity, BV2, NEL-MG, neonatal microglia, and adult microglia at a density of 2 × 10^5^ cells/mL were seeded on a 12-mm coverslip in 24-well cell culture dishes. Each souce of micorglia were treated with 2 μL of red fluorescent latex beads (2 μm, Sigma-Aldrich, St. Louis, MO, USA). NEL-MG showed highest phagocytic activity among them. The data are shown as mean ± standard error of the mean (SEM). Statistical analysis was indicated as a table (below).

**
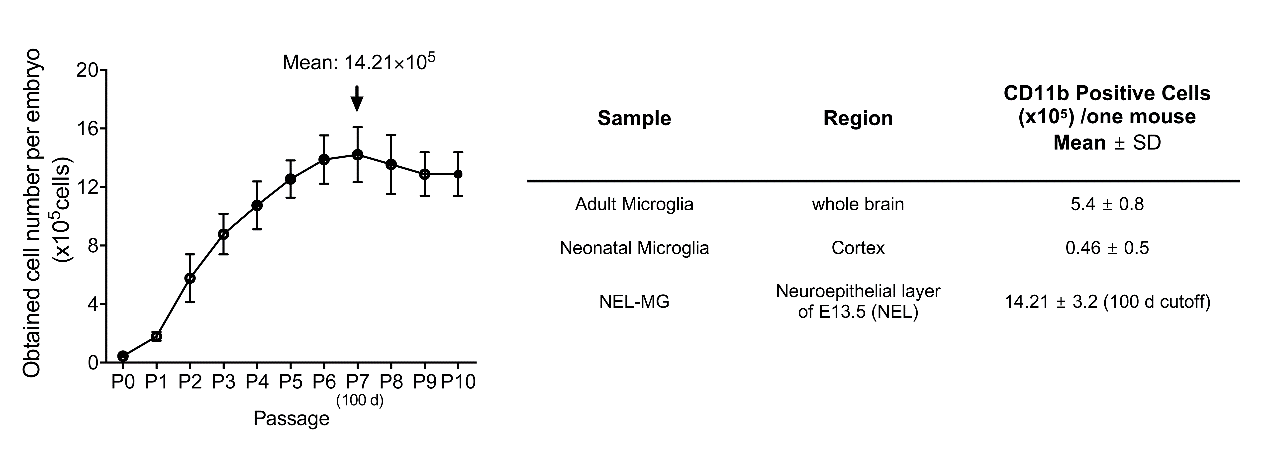
**

**Figure S3. Mass production of microglial cells via NEC passage culture**

Our method produced thirty times the number of microglial cells than that of neonatal microglia when we used a cutoff of 100 days. The data are shown as mean ± standard error of the mean (SEM).

**
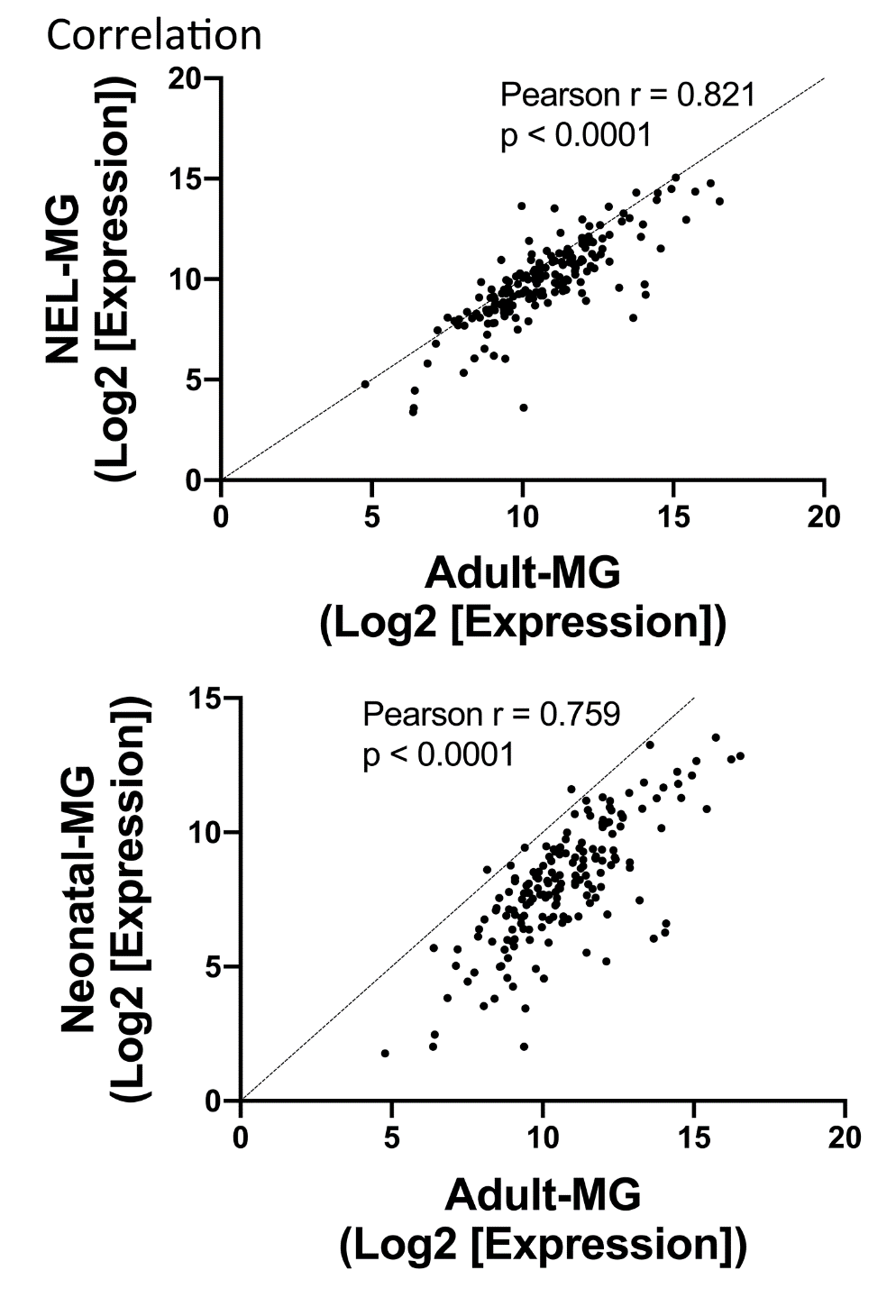
**

**Figure S4. Scatter plot comparison between adult microglia and NEL-MG or neonatal microglia (MG)** Diagonal line indicates no significant difference between the two groups (fold change = 1) and the intensity values are normalized to the log2 transformed expression value.


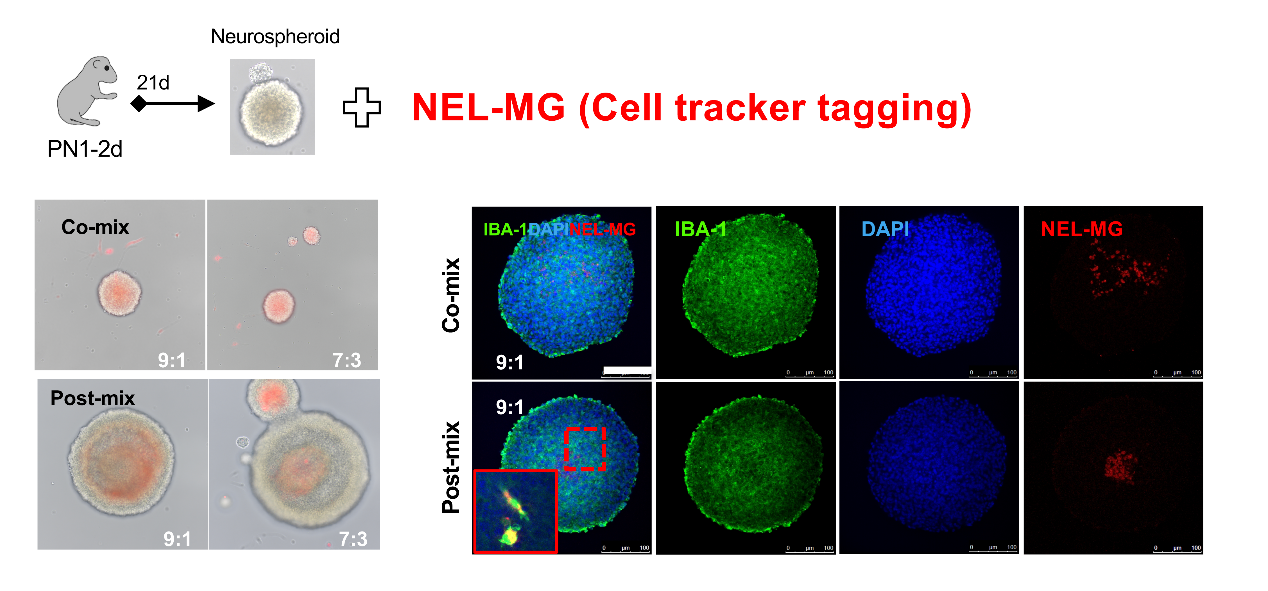


**Figure S5. Mix with neurospheroid**

CellTracker-tagging NEL-MG (red) were mixed with neurospheroids (NS) at different ratios and times. NEL-MG were mixed evenly with NS when we co-mixed them. IBA-1 can label both resident microglia and mixed NEL-MG.

**TABLE S1. LIST OF MICROGLIAL SIGNATURE GENES**

| Ly96 | Clec5a | Itgb5 | Lrrk1 | Ptprm | Inpp4b |
| --- | --- | --- | --- | --- | --- |
| Abca9 | Trim47 | Synj1 | Itga6 | Slc4a2 | Tgfb1 |
| Abcc3 | Socs3 | Gcnt1 | Ltc4s | Tlr6 | Tlr13 |
| Adap2 | Cmklr1 | Rgmb | Acp2 | Scarb2 | Syngr1 |
| Rgs1 | Cmtm6 | Gmfg | Ly86 | Ttc28 | Tlr2 |
| AF251705 | Rock2 | Gna15 | Lyl1 | Plcg2 | Pla2g15 |
| Aif1 | Agmo | Gpr34 | Lyn | Pld4 | Tlr4 |
| Arap3 | Csf1r | H2-DMb1 | Abhd12 | Plek | Tmem119 |
| Arhgap30 | Fos | Egr1 | Lgmn | Prdm1 | Cadm1 |
| Arhgdib | Kcnk13 | Hck | Mafb | Pon3 | Bco2 |
| Atf3 | Csf2rb | Hmha1 | Tmem144 | Psd4 | Map2k1 |
| Tmem206 | Ctsc | Hpgd | Man2b1 | Ptafr | Mras |
| Baz1a | Rapgef5 | Rasgrp3 | Ctss | Scoc | Tnfrsf13b |
| C1qa | Cysltr1 | Hpgds | Mpeg1 | Frmd4b | Tnfrsf1b |
| Sesn1 | Cyth4 | Fcgr1 | Mrc1 | Ptpn6 | Tgfbr2 |
| C1qb | Dennd1c | Cd14 | Ms4a6c | Pycard | Trem2 |
| P4ha1 | Rnf180 | Hvcn1 | Myo1f | Rab3il1 | Tlr7 |
| C1qc | Arhgap22 | Nr3c1 | Naip6 | Rac2 | Trim30a |
| Lrrc3 | Ang | Icam1 | Ncf1 | Ppp1r15a | Tnfrsf11a |
| Cbr2 | Entpd1 | Il21r | Rap1gds1 | Tmem86a | Chst7 |
| Nuak1 | Epsti1 | Irf5 | Ncf2 | Nav2 | Ophn1 |
| Ccdc88b | Lacc1 | Itga9 | Neurl3 | Mef2a | Uba7 |
| Ccr5 | Kctd12 | Itgb2 | P2ry6 | Slc15a3 | Vav1 |
| Basp1 | Fcer1g | Laptm5 | Nfam1 | Slc25a45 | P2ry12 |
| Bhlhe41 | Fcgr2b | Lcp1 | Nfatc1 | Slc7a7 | Mertk |
| Cd33 | Pdgfb | Lpcat2 | Nlrp1b | Pde3b | Gas6 |
| Rtn4rl1 | Ctsf | Il10ra | Pmepa1 | Slco2b1 | Hexb |
| Ccl2 | Fcgr3 | Blnk | Pros1 | Spi1 |  |
| Cd53 | Fcrls | Jun | P2ry13 | Ebf3 |  |
| Ccl4 | Cx3cr1 | Lpxn | Spsb1 | Large |  |

**TABLE S2. PRIMERS INFORMATION**

| *Primer* | sequence |
| --- | --- |
| *Trem2* | *F: TGGGACCTCTCCACCAGTT*  *R: GTGGTGTTGAGGGCTTGG* |
| *iNOS* | *F: CATTGGAAGTGAAGCGTTTCG*  *R: CAGCTGGGCTGTACAAACCTT* |
| *Tnf-a* | *F: GAGTCCGGGCAGGTCTACTTT*  *R: CAGGTCACTGTCCCAGCATCT* |
| *IL-1b* | *F: GGCTGGACTGTTTCTAATGC*  *R: ATGGTTTCTTGTGACCCTGA* |
| *IL-6* | *F: CCACTTCACAAGTCGGAGGCTTA*  *R:GCAAGTGCATCATCGTTGTTCATAC* |
| *CCL3* | *F: CCAAGTCTTCTCAGCGCCAT*  *R: TCCGGCTGTAGGAGAAGCAG* |
